# Use of complementary and alternative medicine in pregnancy and labour pain: a cross-sectional study from turkey

**DOI:** 10.1186/s12906-022-03804-w

**Published:** 2022-12-14

**Authors:** Ruşen Öztürk, Ayşe Emi̇nov, Gül Ertem

**Affiliations:** 1 Ege University, Nursing Faculty, Women Health and Diseases Nursing Department, Izmir, Turkey; 2 Ege University, Faculty of Health Sciences, Graduate Student, Bornova, 35100 Izmir, Turkey

**Keywords:** Complementary medicine, Pregnancy, Preparing for the birth, Symptoms management

## Abstract

**Background:**

This study aimed to determine the prevalence and pattern of complementary and alternative medicine (CAM) use by and attitudes towards CAM of Turkish women during pregnancy and birth.

**Methods:**

This was a descriptive, cross-sectional study that included 260 women who gave birth in a university hospital. The Personal Information Form and Complementary, Alternative and Conventional Medicine Attitudes Scale (CACMAS) were used as data collection tools.

**Results:**

A total of 71.5% of the pregnant women received CAM. Phytotherapy, spiritual meditation and therapeutic touch techniques were the most frequently used techniques. A total of 42.7% women used herbal products. The mean score of the women on the CACMAS scale was 108.37 ± 7.71; this result indicates that pregnant women had a positive attitude. There were significant differences in attitudes according to marital status, education level and place of residence. It was determined that there was a significant difference in the CACMAS scores of the women according to the symptoms experienced during pregnancy, CAM use during pregnancy and the concerns about triggering preterm birth by using CAM methods (*p* < 0.05).

**Conclusion:**

Although the women commonly used CAM methods during pregnancy, the rate of using these methods during birth considerably decreased. Despite such widespread use, pregnant women have concerns about CAM methods. Therefore, health professionals must actively provide counselling on CAM methods for the protection of maternal and infant health.

## Background

Complementary and alternative medicine (CAM) use is prevalent in both developing and developed countries. World Health Organisation reported that more than three-quarters of the world’s population rely on CAM for healthcare [1]. Considering the thoughts of the women in some cultures, it can be believed that CAM is acceptable as natural and safe method, so women evaluate that method as safer option for health problems during their pregnancy [2, 3]. Thus, prevention and treatment of some complications associated with pregnancy may lead to the increased CAM use [1–4]. It has been observed that CAM use during pregnancy and birth varies with a widespread distribution between 1% and 87% [1, 5–7]. Herbal therapies, vitamins, meditation, massage and yoga are among the most frequently used CAM methods [7–10]. Despite the increasing CAM use, the lack of evidence regarding its safety and efficacy on maternal and infant health during pregnancy increases the concerns regarding CAM use during pregnancy by women [1, 3, 4].

Although limited data are available regarding the outcomes after CAM use during pregnancy, there are various pieces of evidence indicating that CAM use can adversely affect pregnancy and birth [1, 11]. Although some CAM methods may cause serious health problems, others are effective and popular strategies that result in several positive outcomes [12], such as assisting with labour preparation and increasing normal birth; aiding in relaxation and psychological well-being as well as retaining control over health decisions [7, 13, 14]. However, these benefits have limited evidence supporting the use of general CAM [15].

Pregnant women may use CAM methods for their own healthcare, autonomy and preference to make their own decisions or possibly for the perception that CAM is natural and not harmful [6, 7, 16]. The fear of teratogenic drugs prescribed during pregnancy and the thought that conventional treatment is not welcomed by the society may cause more frequent use of herbal products by pregnant women [17, 18]. Many herbal products are not sufficiently tested for their safety, particularly during pregnancy. There are many concerns associated with the use of herbal products, such as malformations, in the unborn baby owing to teratogenicity; carcinogenic effects; foetal distress; low birth weight and birth defects; miscarriage or premature birth; the lack of appropriate quality control mechanisms and standardisation for safety and efficacy of certain herbs; potential drug interactions with traditional drugs; uncertainty regarding the purity and safety of plants; contamination and lack of product quality owing to incorrect labelling [2, 9, 19].

Another concern is the birth process, which is a natural and physiological event, which is a milestone in women’s lives. Labour pain can lead to tension, anxiety and fear that negatively affect women’s labour experience [20, 21]. The three main principles of pain relief considered in obstetrics are simplicity, safety and protection of foetal homeostasis. The methods to be applied in labour pain management should reduce pain and increase the satisfaction of the woman with the birth [22]. Breathing techniques, massage, hypnotherapy, reflexology, herbal medicines, homoeopathy, hypnosis, music and magnets as well as acupressure or acupuncture are some of the non-pharmacological pain management techniques [23]. Acupuncture and hypnosis are reported to relieve labour pain [20]. Other methods may play a role in reducing pain; however, the quality of evidence was insufficient and further randomised controlled trials were needed [24]. An effective CAM practice helps in having a normal, non-medicalised birth; developing the quality of care and presenting a women-centred approach. Therefore, health professionals should promote CAM use to support women in labour [25].

Disclosure of CAM use by medical providers is an important issue. A meta-analysis found that the general population had a 33% disclosure rate [26]. The NHIS study reported that 42.3% did not disclose their most used CAM methods [27]. This lack of discussions and disclosures may lead to delay or avoidance in obtaining appropriate treatment and may also endanger maternal and foetal health by interfering with the effect mechanism of a drug [7]. Healthcare staff must be aware of this relatively common practice. They should also have evidence-based information about the effects, side effects and possible risks of using CAM [18]. Turkey has a rooted background in the use of traditional medicine that seems to be boosting in recent years; however, there are limited data available on the prevalence of CAM use among pregnant women in Turkey, and primarily made use of herbal and massage therapies [28, 29]. However, the previous studies include only the pregnancy period and primarily focusing on the different purposes of CAM usage, thus this study aimed to fill these gaps in order to understand the prevalence and pattern of CAM use and attitudes towards CAM of Turkish women during pregnancy and birth.

## Methods

### Study design

This was a descriptive cross-sectional study. The study population comprised women who gave birth in the Gynaecology and Obstetrics Department of Ege University Hospital between September 2019 and September 2020. The population of the study was calculated with the sample formula for a known universe according to 1089 mothers who were expected to give birth at Ege University Hospital in ‘2019–2020’, and it was aimed to reach 285 women with a 95% confidence interval and a 5% type-I error rate. The sample of the study was determined as 285 people using the formula of selection of a known population [30, 31]. A simple random sampling technique was used to select women after determining the sample size; lists of the newborn mother were received from the patient record system of the hospital regularly and then a computer program was used to employ for choosing. Thus, women who are on list were determined using the random number generator program, which is a completely simple random computer-aided randomization program. The researcher made this selection up to reaching 285 postpartum women. Overall, 285 postpartum women were recruited the study; however, a total of 260 mothers were finally included who had fully completed the survey, 25 of the responses were excluded because of the incompleteness of filling the survey and dropping out of the study owing to postpartum complications, resulting in a response rate of 91.2%. The study participants had no serious complications during birth, were between the ages of 18 and 49 years, were literate, had a normal birth at term, knew Turkish, had no mental health problems and agreed to participate in the study. Women who were in intensive care unit, in high dependency unit or were being managed for a severe condition or their baby was in NICU were excluded from the study. Women who were unable to speak or were mentally unstable were also excluded.

### Data collection tools

The questionnaire composed of three sections to collect the data. The Personal Information Form comprised 21 questions; the Complementary Medicine Practices Information Form comprised 15 questions; Complementary, Alternative and Conventional Medicine Attitudes Scale (CACMAS) comprised 27 items.

#### Personal information form (section A)

 The Personal Information Form comprises 11 questions, including demographic and familial characteristics of women (e.g. age, marriage status, educational level, family type, social insurance, occupation, perception of family income, living place etc.) and 10 questions on their obstetric and medical history (e.g. previous pregnancy history, previous birth, pregnancy planning, time between pregnancies, most common complaints).

#### CAM practices information form (Section B)

This form comprised questions on the CAM methods, products and treatments used by women during pregnancy and birth. The methods were defined as CAM according to the criteria of the National Institute of Health National Complementary and Alternative Medicine Centre (https://www.nccih.nih.gov/). The CAM Practices Information Form included 15 questions on perceptions of the benefits and risks of the CAM methods, consultation on using CAM, the reason for using CAM, frequency of CAM use, concerns regarding CAM methods and commonly used methods during pregnancy and labour as well as satisfaction of women with the CAM methods [32, 33].

#### CACMAS (section C)

 The CACMAS was developed by McFadden et al. (2010) to evaluate individuals’ attitudes towards complementary medicine practices, and its Turkish validity and reliability study was conducted by Köse et al. (2018). The CACMAS is a Likert-type scale consisting of 27 items and 3 sub-dimensions. The items are scored between 0 and 7, with 7 indicating ‘strongly agree’ and 0 indicating ‘strongly disagree’. The sub-scales are the intellectual view of complementary medicine, holistic view of health and dissatisfaction with modern medicine. No cut-off points were used, and a higher total score was representative of more favourable attitudes towards CAM. The lowest score that can be obtained on the scale was 7 and the highest score was 189. Cronbach’s alpha coefficient of the scale was found to be 0.808; Cronbach’s alpha values of the sub-scales ranged between 0.683 and 0.825 [17, 34] and in this study, it was calculated to be 0.81.

A and B sections of the questionnaire were developed after a detailed review of previous international and national studies [17, 19, 32, 33]. The Turkish validity and reliability of the CASMAS scale has been assessed in a previous study [17]. A pilot study was conducted with 10 women, who gave birth shortly before the study, to evaluate the comprehensibility of the questions and contents in the questionnaire. Some adjustments were made to make it more comprehensible and simpler to complete after pretesting.

#### Ethical statement

The Board of Ege University Clinical Research Ethics Committee approved this study (Approval no: E.321,347; 16 September 2019). The participants were informed about the purpose of this study, benefits provided by the study and the time that they need to allocate for the study before interviews. Written consent was obtained from all participants. The questionnaire was completed by the participants themselves.

#### Data analysis

Data analysis was performed using the SPSS 22.0 programme (IBM Corp., Armonk, NY). Kolmogorov–Smirnov and Shapiro–Wilk W-tests were used to determine the normal distribution of the data. It was shown that the data were non- normally distributed. Descriptive statistics tests (number, percentage, standard deviation and mean) were used to compare the distributions of the data, and the Mann–Whitney U test and Kruskal–Wallis test were used to compare non-normally distributed variables. Multiple linear regression analyses were carried out for the identification of determinants (categorical independent variables) associated with CAMCAS scores (continuous dependent variables). All findings were evaluated at a 95% confidence interval and a *p*-value of < 0.05. Vitamins were excluded from the analysis because folic acid, iron supplements and calcium supplements were commonly recommended by doctors, and the majority of women were taking them.

## Results

The median age of the pregnant women was 29 ± 4.76 years (minimum = 19 maximum = 43); 96.5% of them were married and 3.5% were single; 41% of them were high school graduates, 30.4% had primary education and 16.9% went to the university; 11.5% were literate. While 55% of them had no paid employment, 25.0% were officers, 11.2% were workers and 8.8% were self-employed (Table 1). The median gestational week of the women was 38 ± 1.33; their median pregnancy number was 2 ± 1.42; 42% of them were in their first pregnancy; 50% had given birth before; 79% had a planned pregnancy and 42% had > 2 years of gestational intervals (Table 2).

**Table 1:** Distribution of the Sociodemographic Characteristics of Pregnant Women and the Alternative and Conventional Medicine Attitudes Scale (CACMAS)

Sociodemographic Characteristics	N	%		*U/X* ^*2*a^	*P* value
**Age**					
18–30	105		56.5	U = 4136.000	0.833
> 30	81		43.5		
**Marital status**					
Married	251	96.5		**U = 665.000**	0.37*
Single^b^	9	3.5			
**Educational level**					
Literate^b^	30	11.5			
Primary education	79	30.4		***X***^**2**^ **= 8.128**	0.44*
High school^b^	107	41.2			
> University	44	16.9			
**Family type**					
Nuclear family	221	85.0			
Extended family	39	15.0		*U* = 4138.000	0.724
**Social insurance**					
Yes	247	95.0			
No	13	5.0		1244.500	0.177
**Profession**					
Not in paid employment	143	55.0			
Officer	65	25.0		*X*^2^ **=** 1.215	0.749
Worker	29	11.2			
Self-employment	23	8.8			
**Perception of family income**					
Bad (income < expense)	27	10.4		*U* = 2779.000	0.498
Medium (income = expense)	233	89.6			
**Where they live**					
Province	90	34.6		***X***^**2**^ **= 9.42**	0.009*
District	143	55.0			
Village^b^	27	10.4			

*P < 0.05

^a^*U*= Mann-Whitney *U* test; *X*^2^ = Kruskal Wallis-H test

^b^Significantly higher

**Table 2:** Distribution of Obstetric Characteristics of Pregnant Women and the Alternative and Conventional Medicine Attitudes Scale (CACMAS)

Obstetric Characteristics	N	%	*U/X*^*2*^^a^	*P* value
**Previous pregnancy history**				
Yes	151	58.1	U = 7251.500	0.120
No	109	41.9		
**Previous birth**				
Yes	129	49.6	U = 7480.500	0.133
No	131	50.4		
**Planned pregnancy**				
Yes	205	78.8		
No	52	20.0	U = 0.710	0.701
With treatment	3	1.2		
**Time between pregnancies**				
< 2 years	44	16.9		
> 2 years	107	41.2	***X***^**2**^ **=** 3.451	0.178
Not applicable	109	41.9		
**Most common complaints**				
Back, neck or hip pain^b^	40	15.4		
Reflux or heartburn	21	8.1	***X***^**2**^ **= 45.613**	< 0.001*
Nausea and vomiting	49	18.8		
Headache or migraine	22	8.5		
Sleep problems	18	6.9		
Vaginal bleeding	16	6.2		
Tiredness	34	13.1		
Hypertension	14	5.4		
Other (GDM, varicose veins, haemorrhoids, anaemia, excess weight gain, UTI)	32	12.3		
No complaints	14	5.4		

*P < 0.05

^a^U= Mann-Whitney U test; *X*^2^ = Kruskal Wallis-H test

^b^Significantly higher

CAM use (before getting pregnant) during their lifetime was 21.2%, but it increased to 71.5% during pregnancy. Phytotherapy (31.2%), meditation (25.3%) and therapeutic touch (11.8%) were reported to be the most frequently used CAM methods. A total of 42.7% women used herbal products during pregnancy. It was stated that these products were mostly used for relaxation (46.8%), blood pressure problems (16.7%) and foetal health (13.4%). It was reported that 55.9% applied the methods on their own. Of these, 84% were reported to perceive benefits, 91% did not experience any harm, 71% recommended the methods to others and 72% stated that they were worried that CAM methods might lead to threatened preterm birth. During their pregnancy, the mean number of CAM methods applied by the women was 3.059 ± 2.29. Although only 13% of them applied back massage, the mean pain level was found to be significantly lower in those who used this method (6.79 ± 1.50) (Table 3).

**Table 3:** Distribution of the Characteristics of the Use of CAM by Pregnant Women and the Alternative and Conventional Medicine Attitudes Scale (CACMAS)

Experience with CAM Methods	N	%	*U/X*^*2*^^c^	*P* value
**Previous CAM usage**				
Yes	55	21.2	U = 5175.500	*p* = 0.462
No	205	78.8		
**Use of CAM methods during pregnancy**				
Yes*	181	71,5	**U = 48.566**	***p*** < 0.001*
No	74	28.5		
**Method used during pregnancy**^**a**^				
Phytotherapy	58	31.2		
Spiritual meditation	47	25,3		
Therapeutic touch	22	11.8		
Art therapy	13	7,0		
Acupuncture	13	7,0		
Body and foot massage	11	5,9		
Music therapy	8	4,3		
Pilates	7	3,8		
Yoga	5	2,7		
Hypnosis	2	1,1		
**Usage of herbal products**^**a**^				
No	149	57.3		
Linden	20	7.7		
Ginger	23	8.8		
Honey	16	6.2		
Turmeric	10	3.8		
Mint and lemon	17	6.5		
Herbal medicine for nausea	9	3.5		
Raisin	16	6.2		
**Reason for use of CAM methods**^**b**^				
For nausea, vomiting	18	10.5		
For waist and back pain	12	7.5	*X*^2^ = 8.879	*p* = 0.180
For foetal health and healthy pregnancy	25	13.4		
To keep time active	9	5.8		
To relax	87	46.9		
Blood pressure	30	16.7		
**Consulting about CAM methods**^**b**^				
Relatives and Friends	25	13.4		
Doctor	17	9.1	*X*^2^ = 4.887	*p* = 0.299
Nurse	1	0.5		
Self usage	104	55.9		
Other practitioners	39	21.0		
**Finding benefits of CAM usage**^**b**^				
Yes	156	84.4		
No	8	4.3	*X*^2^ = 1.039	*p* = 0.595
Partially	19	11.3		
**Experiencing harm**^**b**^				
Yes	1	0.5	X^2=^5.000	*p* = 0.082
No	166	91.4		
Partially	15	8.1		
**Recommended the methods**^**b**^				
Yes	130	71.0	*X*^2=^0.525	*p* = 0.769
No	21	11.3		
Some	31	17.7		
**Concern about triggering threatened preterm delivery**				
Yes*	187	71.9	***X***^**2**^ = **12.393**	*p* = **0.002**
No	62	23.8		
Not Sure	11	4.2		
**Methods used during birth**				
Epidural Birth	55	21.1	*X*^2^ = 0.593	*p* = 0.774
Back massage	34	13.2		
No	171	65.7		
**Pain level at birth**				
Using method	89	6.79 ± 1.50	(minimum 2, maximum10)	
Not using method	171	8.51 ± 0.075	(minimum 5, maximum 10)	
Total	260	7.83 ± 1.39	(minimum 2, maximum 10)	

*P < 0.005

^a^More than 1 answer was given

^b^Calculated over those using CAM during pregnancy (*n* = 181)

^c^U = Mann-Whitney U test; *X*^2^ = Kruskal Wallis-H test

The mean score of pregnant women on the whole CACMAS was 108.37 ± 7.71. The scores on the sub-scales of the intellectual view to complementary medicine, dissatisfaction with modern medicine and holistic view of health were found to be 33.46 ± 2.95, 33.04 ± 4.49 and 41.85 ± 4.75, respectively. A significant difference was found between the total score and marital status, education level and place of residence. Pregnant women who were single, literate, studied until high school and lived in a village were found to have significantly higher CACMAS score. Significant differences were found in the mean CACMAS scores with respect to the symptoms experienced during pregnancy. Women who had back, neck or hip pain complaints and who used the CAM methods during pregnancy had higher CASMAS score. Women who were concerned about triggering problems such as preterm birth with CAM use had lower CACMAS score (*p* < 0.05) (Table 3).

The regression analysis was used to explain the factors affecting the women’s CACMAS scores. The potential influencing factors showing statistically significant associations were selected in the multivariate regression analysis. The predictive power of the regression model calculated using the Backward selection method was 7.9%. (*R*^2^:0.079, F: 3.263, Durbin-Watson: 1.677, *p* = 0.004). The independent variables were the factors significantly associated with CACMAS score (the dependent variable in this case) in the univariate analyses including residency, marital status, education level and concern about triggering problems. Results showed that pregnant women who were single (β:5.286, p:0.041 ), graduated at high school (β:2.185, p:0.023), lived in a village (β:2.185, p:0.036) and women who didn’t concern about triggering problems due to using CAM (β:-2.764, p:0.009) have higher CACMAS scores (Table 4).

**Table 4:** Multivariable linear regression analysis showing independent variables associated with the CACMAS score in women

Variables^a,b^	Unstandardized Coefficients		Standardized Coefficients	t	Sig.
	B	Std. Error	Beta		
(Constant)	109.293	1.016		107.540	**0.000**
**Marital status** Single	5.286	2.578	0.126	2.050	**0.041**
**Residency** Village	2.142	1.537	0.085	1.393	**0.036**
**Concern about triggering threatened preterm delivery** Yes	-2.764	1.053	− 0.161	-2.625	**0.009**
**Education level** High school	2.185	0.953	0.139	2.292	**0.023**

*R*^2^: 0.079, F: 3.263, Durbin-Watson: 1.677, *p* = 0.004

^a^ Univariate variables with *P* values < 0.05 were entered into the multiple linear regression

^b^ Nominal variables were entered into analysis using dummy coding

## Discussion

CAM use is reported to be common worldwide during pregnancy. It was revealed in a recent survey that 69% of women in the United States, 57% of women in England, 51% of women in Germany, 52% of women in Australia and 70% of women in Bangladesh use CAM methods [7, 16, 19, 32, 35]. This study indicated that the rate of CAM use increased to 71.5% during pregnancy and had a higher prevalence than previously reported. Various factors such as differences in culture and ethnicity, sociodemographic factors, the use of health services and the difference in CAM treatments in a local area, lack of or inconsistent CAM definition and study design were reported to be responsible for these differences in the prevalence of CAM use [7, 32, 35, 36]. When compared with the international literature, some differences regarding the various CAM use methods of pregnant women were found as well. Popular CAM methods during pregnancy include nutritional supplements, herbal medicines, vitamins, yoga and meditation, massage, relaxation methods, acupuncture and homoeopathy [3, 7, 9, 16]. This study determined that phytotherapy, spiritual meditation and therapeutic touch were the most commonly used methods. These results were quite similar to the findings of Swan and Strouss et al. [37, 38]. Other studies conducted among the general population in our country report that herbal products and religious rituals are the most widely used CAM [39, 40]. This is a very important finding because it shows that the approach towards culturally widely used CAM applications continues during pregnancy as well. However, this should be interpreted with caution because of the potential impact of culture and ethnicity. These require further studies to examine and explore previous CAM use as well as cultural practice and belief and health outcomes of pregnant women.

The mean score of pregnant women on the whole CACMAS was 108.37 ± 7.71; the scores on the sub-scales of the intellectual view to complementary medicine, dissatisfaction with modern medicine and holistic view of health were found to be 33.46 ± 2.95, 33.04 ± 4.49 and 41.85 ± 4.75, respectively. Pregnant women were found to have positive attitudes towards CAM methods in general. Socioeconomic status, health insurance coverage, religion, education, income, parity, place of residence, the use of herbal medicines for other conditions, old age, employment status, marital status, personal attitudes towards both CAM and birth, CAM use before pregnancy, chronic disease/medication, cultural norms and health beliefs were reported to influence women with respect to CAM use [3, 8, 36, 41]. In our study, pregnant women particularly those who lived in villages, were single, had a high school level of education, and had no concerns about triggering problems due to using CAM had a positive association between the CACMAS score.

And also, significant differences were observed in this study between the women’s scores on the CACMAS with respect to symptoms experienced during pregnancy. Thus, this study’s result can be interpreted as pregnant women who have pregnancy complaints have positive attitudes towards CAM methods and this could be the reason for increased CAM use. The concern and uncertainty of the risk of preterm birth can cause negative attitudes towards CAM.

Some studies reported that between 7% and 82% of pregnant women use herbal remedies or other natural health products. Different herbal medicines are used by different ethnic groups because diverse cultural backgrounds and traditional beliefs [2, 11, 36, 41].  The most used herbs during pregnancy are chamomile, ginger, blueberry, mint, cranberry, valerian, raspberry, echinacea, black cumin, lemon tea, anise, cinnamon, castor oil, prune and mustard oil and raspberry leaves [32, 35, 37, 41, 42]. Ginger, honey, lime, mint-lemon and raisins were most commonly used by the participants in this study. Thus, this reveals that herbs differ among different cultures and countries [41]. Women appear to be interested in herbal remedies because of their preference to use a natural substance. Women believe that ‘natural’ implies ‘safe’ and have the perception that these products are safer than other drugs and are associated with no risk [9, 19, 32, 38, 41]. Many women are unaware of their side effects and generally self-prescribe herbal medicines because of the belief that they are harmless during pregnancy [19, 32]. Although some herbal medicines are reported to be useful and have low side effects, there is no sufficient evidence to prove their safety and efficacy during pregnancy [19, 41]. Thus, providers should inquire about the routine CAM use specific for their trimester and complaints faced by the pregnant women.

The following are the most frequently reported reasons for CAM use: its affordability, accessibility, effectiveness, safety and naturality; familial and cultural traditions; dissatisfaction with modern medicine; its ability to improve health conditions during pregnancy; prevention of general diseases and complications; protection of foetus health; its ability to enhance immunity and relaxation as well as maintain well-being and preparing the uterus for birth [7, 9,  32, 35].  Similar results were observed in this study: CAM methods were used for relaxation, maintenance of the health of the foetus as well as pregnant woman and alleviation of symptoms. Although the reason for CAM use to prepare for childbirth and to facilitate it is common [32, 41], this reason was not specified in this study. These results are also reflected in the CAM use during birth, with only 13% who applied back massage during labour. CAM use during birth helps women to perceive birth as a natural event, decreases their fear and anxiety levels, increases their ability to cope with labour pains, increases their self-confidence and provides them a more positive birth experience [4]. Pregnant women do not benefit enough from the application of CAM therapies to facilitate the birth because health professionals use or offer limited CAM practices in a community/birth centre. In this context, the crucial role played by the providers with respect to CAM practices and consultation at labour and pregnancy is worth mentioning.

There is little evidence to support CAM use during pregnancy, which raises questions of its potential risks and possible benefits [16, 41]. Most pregnant women in this study reported that they perceived benefits of CAM use, did not experience any harm and recommended CAM to others. However, majority of them were also worried that CAM use might lead to preterm birth. In a study conducted in Iran, 83.7% of the pregnant women were satisfied with CAM use [32]. 8% of the pregnant women in a study conducted in Bangladesh experienced side effects [35]. In a study conducted in Palestine, 91.7% of the women found these treatments beneficial, and 99.2% of them did not report side effects [41]. These results are quite similar with our study findings. Although high use and high utility rates have been reported, still nearly three-quarters of pregnant women fear that CAM methods may cause preterm birth. This paradoxical situation indicates that most users were not aware of the safety, efficacy or potential drug interactions [36, 41]. It was observed that women mostly do not inform their midwives or obstetricians about their CAM use; health professionals do not question the use of CAM; they use CAM methods on their own [7, 16, 19, 42]. Approximately 56% of the women in this study reported that they used CAM on their own without consulting. Therefore, the concerns about CAM use by pregnant women without the knowledge or contribution of healthcare professionals should be underlined. Thus, health professionals play a key role in maternity care that requires closer attention. They should increase their knowledge to allow women who use CAM and those providing care to women during pregnancy and birth to be fully informed [7, 16, 19, 23, 38]. Moreover, the results of this study provide a basis for CAM use in pregnancy as well as provide an understanding of positive attitude toward CAM use by pregnant women in Turkey where traditional practice and usage is common. These outcomes indicate the risk for pregnant women that are more likely to use methods and show the importance of counselling about CAM use to prevent its side effects. Thus, pregnant women should be counselled comprehensively on CAM which can eventually lead to the use of methods that do not compromise maternal and fetal health.

### Limitations

This study has some limitations. First, it was conducted in a single hospital with random sampling. Therefore, the results are likely to be valid only for the local population. Further studies must be conducted over a wide area, including population groups that represent the regional diversity in Turkey. But the study was carried out in a city with a developed education and economic level in Turkey. These rates reflect a certain part of the society, but even if the results of the study are carried out in a certain region, the results of the study show that the use of CAM is significantly higher due to the low level of education and living in the village due to high migration ratein this region. Therefore, the widespread use of CAM during pregnancy in one of the most developed cities of the country predicts that the study will be widely used throughout the country and may even be used at a higher rate in underdeveloped regions.

A major limitation of this study was the use of postnatal data collection to assess CAM use throughout the pregnancy. The study did not question CAM use for symptoms specific to the trimester. Questioning regarding this in future prospective studies that follow up on a weekly or monthly basis could provide more reliable and valid data. In the study, another limitation might be that complications in the mother and baby were excluded from the study. Given the association of CAMS with complications, these results may be important. However, due to the nature of the clinical survey, in case of complications in the mother and baby, reliable and effective answers to the questions of the survey cannot be obtained.

## Conclusions

In conclusion, high rates of CAM use, particularly the use of phytotherapy, spiritual meditation, and therapeutic touch, during pregnancy were observed, and these methods are highly appreciated. Almost half of the pregnant women in this study reported that they consumed herbal products during their pregnancy. We found had a positive association with CACMAS score including residency, marital status, education level, and having no concern about triggering problems, so providers should take into account these factors as potential CAM users. Thus, the results showed that the concern regarding preterm birth revealed the lack of knowledge among CAM users. Also, the self-use of CAM by pregnant women was particularly alarming because of potential safety issues. Therefore, in terms of the prevalence of CAM use with an emphasis on outcomes, there is an urgent need for enhanced knowledge regarding CAM practice in both pregnant women and health care providers.

## Data Availability

The datasets used and/or analysed during the current study are available from the corresponding author on reasonable request.

## Abbreviations

CACMAS: Complementary, Alternative and Conventional Medicine Attitudes Scale
CAM: Complementary and alternative medicine
